# Sensitivity Analysis and Optimal Control of Anthroponotic Cutaneous Leishmania

**DOI:** 10.1371/journal.pone.0160513

**Published:** 2016-08-09

**Authors:** Muhammad Zamir, Gul Zaman, Ali Saleh Alshomrani

**Affiliations:** 1 Department of Mathematics, University of Science and Technology, Bannu, Khyber Pakhtunkhwa, Pakistan; 2 Department of Mathematics, University of Malakand Lower Dir, Khyber Pakhtunkhwa, Pakistan; 3 Department of Mathematics, Faculty of Science, King Abdul Aziz University, Jeddah, Saudi Arabia; National Centre For Cell Science, INDIA

## Abstract

This paper is focused on the transmission dynamics and optimal control of *Anthroponotic Cutaneous Leishmania*. The threshold condition *R*_0_ for initial transmission of infection is obtained by next generation method. Biological sense of the threshold condition is investigated and discussed in detail. The sensitivity analysis of the reproduction number is presented and the most sensitive parameters are high lighted. On the basis of sensitivity analysis, some control strategies are introduced in the model. These strategies positively reduce the effect of the parameters with high sensitivity indices, on the initial transmission. Finally, an optimal control strategy is presented by taking into account the cost associated with control strategies. It is also shown that an optimal control exists for the proposed control problem. The goal of optimal control problem is to minimize, the cost associated with control strategies and the chances of infectious humans, exposed humans and vector population to become infected. Numerical simulations are carried out with the help of Runge-Kutta fourth order procedure.

## Introduction

*Anthroponotic cutaneous leishmaniasis* (ACL) is an infectious disease caused by Leishmina Tropica. The parasite is transmitted by the sand fly Phlebotomus sergenti; about 2–3mm long, and is sandy-colored. These flies are blood-feeding and breed in forest areas, caves, or the burrows of small rodents. The sand fly latent period is assumed roughly to be 3–7 days [1]. Leishmaniasis is a group of infectious diseases. utaneous leishmaniasis(ACL), Muco-cutaneous leishmaniasis(MCL), Visceral leishmaniasis (VL) or kala-azar, and (PKDL) Post-kala-azar dermal leishmaniasis are four main clinical syndromes of Leishmaniasis [2, 3].

Cutaneous leishmaniasis (*ACl*, *ZCL*) is the most common form of leishmaniasis. The incubation period of Cutaneous leishmaniasis is two to eight weeks, however, in some cases longer periods have been reported. Leishmania major is the causative agent of human Cutaneous Leishmaniasis. Recovery from infection of Leishmania major, results in persistence of parasites at the primary infection site. Which causes long-lasting immunity to reinfection. Vaccination with killed parasites or recombinant proteins induces only short-term protection [4, 5]. Cross-immunity experiments between different species of leishmania is discussed in [6].

In Pakistan, *ACL* has the widest distribution, occurring in urban areas of Punjab (Multan), Balochistan (Quetta) and in the Northern Areas and Azad Kashmir. Cases of ACL are being increasingly reported from towns of Khyber Pakhtunkhwa. In 1997, a large outbreak of ACL was reported from Timargara refugee camp in Khyber Pakhtunkhw [7].

Mathematical model plays an important role in analyzing the transmission dynamics and control of infectious diseases. The formulation process of a mathematical model elucidates assumptions, parameters and variables. Chaves et al. [8] presented a mathematical model of American Cutaneous Leishmaniasis. They discussed the threshold conditions for infection persistence of the disease. In their recent work, Chaves et al. investigated the effect of deforestation on vector borne diseases [8, 9]. Das et al. studied the effect of delay on the dynamics of Cutaneous Leishmaniasis [10]. Calzada et al. investigated the compositional changes that occur in sand fly after the use of thermal fogging [11]. Chaves [12], Bacaer and Guernaoui [13] discussed the seasonal models of Cutaneous Leishmaniasis. Several strategies are adopted to control the transmission and eradication of different infectious diseases. However, it has been always a challenge to establish a balance between the cost and control of a strategy. Optimal control theory plays extra ordinary role in keeping the minimum cost for maximum efficiency of the control strategy. The optimal controls of several epidemic models are described in [14–17].

In this paper, we present a mathematical model for the transmission dynamics and optimal control of *Anthroponotic cutaneous leishmaniasis*. The threshold condition *R*_0_ for initial transmission of infection is obtained by using Next generation method. We investigated and discussed in detail different scenario of biological sense of the threshold condition. The sensitivity analysis of the reproduction number is presented and the most sensitive parameters are high lighted. On the basis of sensitivity analysis, some control strategies are introduced in the model. These strategies reduce the effect of the parameters with high sensitivity indices, on the initial transmission. The model will then be used to determine the cost-effective strategies for eradicating the disease transmission. In order to do this, an optimal control strategy is presented by taking into account the cost associated with control strategies. It is also shown that an optimal control exists for the proposed control problem. The goal of optimal control problem is to minimize, the cost associated with control strategies and the chances of infectious humans, exposed humans and vector population to become infected. Numerical simulations are carried out with the help of Runge-Kutta fourth order procedure. To address the effect of these parameters, we introduce some control variables in the model.

The paper is organized as follows. A mathematical model of the interaction between the human and vector is presented in Section 2. Section 3 represents mathematical and sensitivity analysis of reproduction number *R*_0_. Section 4 is concerned existence of control strategies. In section 5, the optimality system is numerically solved. The discussion and conclusion of the work is presented in section 6.

## Model Formulation

In this section, we present a formulation of mathematical model which represents the Anthroponotic Cutaneous Leishmania infection in a community. The human population consists of four subclasses, *S*_*h*_(*t*) represents the class of susceptible human, *E*_*h*_(*t*) is the *Cl*-latent class of human, *I*_*h*_(*t*) is the human class of infectious with *Cl*, *R*_*h*_(*t*) is the human class recovered individuals from *Cl* at time *t*. Thus the total human population *N*_*h*_(*t*) is *N*_*h*_(*t*) = *S*_*h*_(*t*) + *E*_*h*_(*t*) + *I*_*h*_(*t*) + *R*_*h*_(*t*).

**Fig 1:**
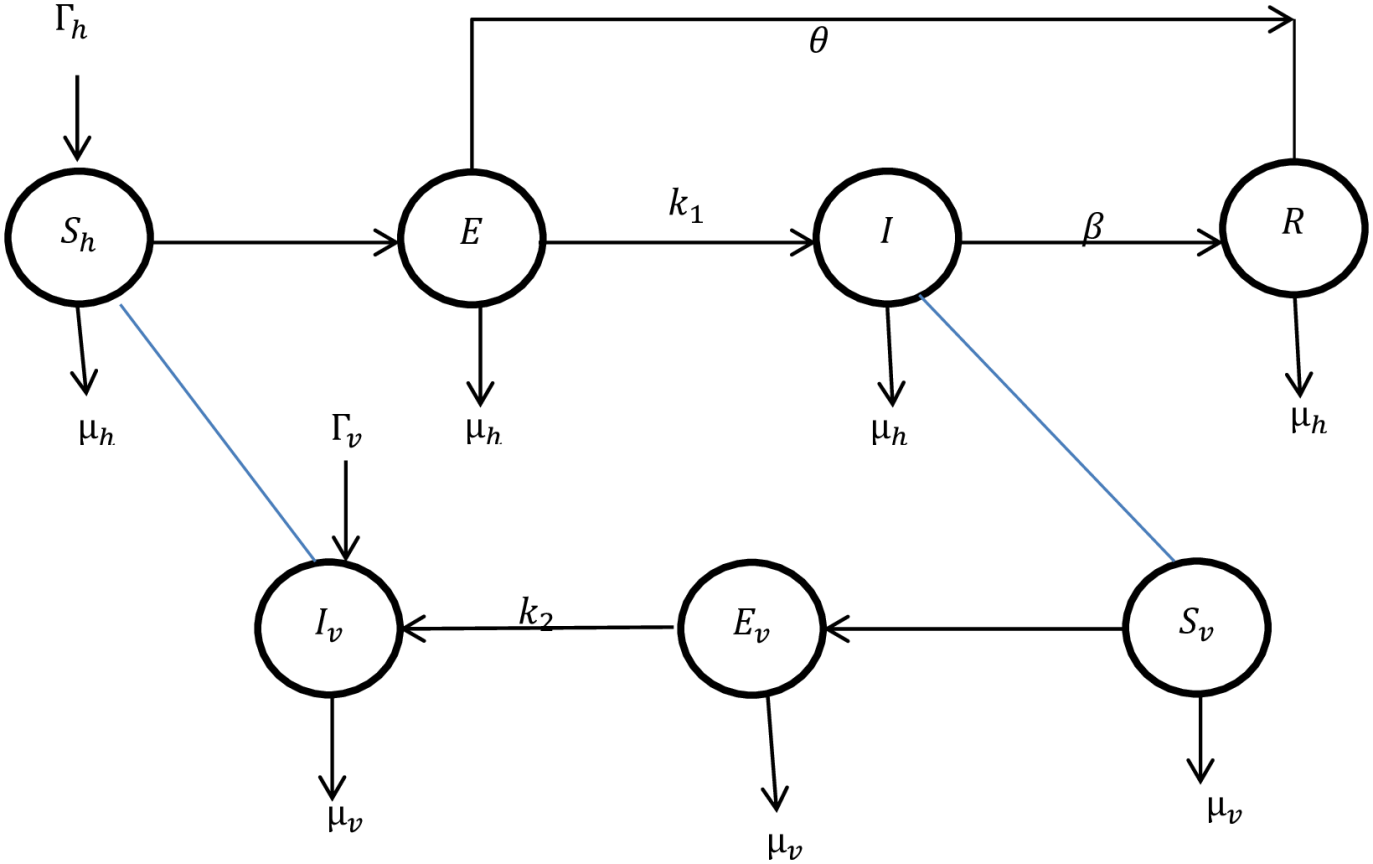
The graph represents the transmission dynamics of the each individual in the model.

The vector population is divided into three sub-classes, *S*_*v*_(*t*) represents the susceptible vector, *E*_*v*_(*t*) is the exposed vector and *I*_*v*_(*t*) infected vector. Total vector population *N*_*v*_ is *N*_*v*_(*t*) = *S*_*v*_(*t*) + *E*_*v*_(*t*) + *I*_*v*_(*t*). The flow chart shows the transmission dynamics of the each individual in the model given in Fig 1. Humans are recruited at a constant per capita rate *Γ*_*h*_ to the susceptible class *S*_*h*_. The susceptible humans after being bitten by infected sand fly, are infected at the rate $\frac{ab_{1}I_{v}S_{h}}{N_{h}}$, where *a* is sand fly biting rate and *b*_1_ is the transmission probability of *ACL* to human from sand fly [18]. The exposed humans are recovered at the rate *θ* without becoming infected and the rest of the exposed humans after completing latency period, get infectious at the rate *k*_1_. Some of the infected humans are recovered naturally at the rate *β*. Sandflies after contact with infected humans are infected at the rate $\frac{ac_{1}IS_{v}}{N_{h}}$, where *c*_1_ is the transmission probability of *ACL* from human to sand fly. After completing incubation period, the exposed sand flies get infectious at the rate *k*_2_. *μ*_*h*_ and *μ*_*v*_ are natural mortality rates of human and sandflies, respectively. The dynamical system for human, reservoir and vector population is given by:

$$\begin{array}{c} \left\{\begin{array}{c} \dot{S_{h}}\left(t\right)=\Gamma_{h}-\frac{ab_{1}I_{v}\left(t\right)S_{h}\left(t\right)}{Nh(t)}-\mu_{h}S_{h}\left(t\right),\\ \dot{E_{h}}\left(t\right)=\frac{ab_{1}I_{v}S_{h}\left(t\right)}{Nh(t)}-\left(k_{1}+\theta +\mu_{h}\right)E_{h}\left(t\right),\\ \dot{I_{h}}\left(t\right)=k_{1}E_{h}\left(t\right)-\left(\beta +\mu_{h}\right)I_{h}\left(t\right),\\ \dot{R_{h}}\left(t\right)=\theta E_{h}\left(t\right)+\beta I_{h}\left(t\right)-\mu_{h}R_{h}\left(t\right),\\ \dot{S_{v}}\left(t\right)=\Gamma_{v}-\frac{ac_{1}I_{h}\left(t\right)S_{v}\left(t\right)}{Nh(t)}-\mu_{v}S_{v}\left(t\right),\\ \dot{E_{v}}\left(t\right)=\frac{ac_{1}I\left(t\right)S_{v}\left(t\right)}{Nh(t)}-\left(\mu_{v}+k_{2}\right)E_{v}\left(t\right),\\ \dot{I_{v}}=k_{2}E_{v}\left(t\right)-\mu_{v}I_{v}\left(t\right). \end{array}\right. \end{array}$$ (1)

## Analysis of the Model

In this section, we study the stability analysis of the proposed model.

### Invariant Region

We assume all the parameters to be nonnegative. The model is concerned with living population, therefore the state variables are assumed to be nonnegative at time *t* = 0. The dynamic of overall population is given by the following differential equations:

$$\begin{array}{ccc} {\dot{N}}_{h}\left(t\right) & = & \Gamma_{h}-\mu_{h}N_{h}\left(t\right), \end{array}$$ (2)

$$\begin{array}{ccc} {\dot{N}}_{v}\left(t\right) & = & \Gamma_{v}-\mu_{v}N_{v}\left(t\right). \end{array}$$ (3)

The non-negative equilibrium of the above equations are

$$\begin{array}{c} N_{h}=\frac{\Gamma_{h}}{\mu_{h}},\;N_{v}=\frac{\Gamma_{v}}{\mu_{v}}. \end{array}$$

Let us consider the biological feasible region Ψ is given by

$$\begin{array}{c} \Psi =\left[\left(S_{h},E,I,R,S_{v},E_{v},I_{v}\right)\in R_{+}^{7}\;,N_{h}\leq \frac{\Gamma_{h}}{\mu_{h}};N_{v}\leq \frac{\Gamma_{v}}{\mu_{v}}\right]. \end{array}$$

By solving Eq (2), we obtain

$$\begin{array}{c} N_{h}=N_{h}\left(0\right)e^{-\mu_{h}t}+\frac{\Gamma_{h}}{\mu_{h}}\left(1-e^{-\mu_{h}t}\right). \end{array}$$

So

$$\begin{array}{c} N_{h}\to \frac{\Gamma_{h}}{\mu_{h}}\;\;as\;\;t\to \infty . \end{array}$$

Similarly we solve Eq (3) to obtain

$$\begin{array}{c} \;N_{v}\to \frac{\Gamma_{v}}{\mu_{v}}\;\;as\;\;\;t\to \infty . \end{array}$$

Hence Ψ is positively invariant domain, and the model is epidemiologically and mathematically well posed.

### Reproduction Number

The number of secondary infection that occurs in completely susceptible population if we introduce an infectious individual to the population, is called reproduction number *R*_0_ [19]. This epidemiological measurement (number) is widely used to measure the transmission potential of the disease in susceptible population [20]. Reproduction number measures the initial disease transmission in population dynamics. The disease can invade the susceptible population and persist if *R*_0_ > 1. The disease dies out and does not spread if *R*_0_ < 1 [21]. To find the basic reproduction number, we use the Next generation method see for detail [22]. *R*_0_ = *ρ*(−*FV*^−1^), where *ρ* is spectral radius. In the absence of exposed human and vector, our model becomes

$$\begin{array}{c} f=\left(\begin{array}{c} f_{1}\\ f_{2}\\ f_{3}\\ f_{4} \end{array}\right)=\left(\begin{array}{c} \frac{ab_{1}I_{v}S_{h}}{N_{h}}\\ 0\\ \frac{ac_{1}IS_{v}}{N_{h}}\\ 0 \end{array}\right), \end{array}$$

and

$$\begin{array}{c} F=\left(\begin{array}{cccc} F_{11} & F_{12} & F_{13} & F_{14}\\ F_{21} & F_{22} & F_{23} & F_{24}\\ F_{31} & F_{32} & F_{33} & F_{34}\\ F_{41} & F_{42} & F_{43} & F_{44} \end{array}\right)={\left(\begin{array}{cccc} 0 & 0 & 0 & ab_{1}\\ 0 & 0 & 0 & 0\\ 0 & \frac{ac_{1}\Gamma_{v}\mu_{h}}{\Gamma_{h}\mu_{v}} & 0 & 0\\ 0 & 0 & 0 & 0 \end{array}\right)}_{\left(DFE\right)}. \end{array}$$

$$\begin{array}{c} v=\left(\begin{array}{c} v_{1}\\ v_{2}\\ v_{3}\\ v_{4} \end{array}\right)=\left(\begin{array}{c} -a_{1}E\\ k_{1}E-a_{2}I\\ -a_{3}E_{v}\\ k_{2}E_{v}-\mu_{v}I_{v} \end{array}\right) \end{array}$$

$$\begin{array}{c} V=\left(\begin{array}{cccc} V_{11} & V_{12} & V_{13} & V_{14}\\ V_{21} & V_{22} & V_{23} & V_{24}\\ V_{31} & V_{32} & V_{33} & V_{34}\\ V_{41} & V_{42} & V_{43} & V_{44} \end{array}\right)={\left(\begin{array}{cccc} -a_{1} & 0 & 0 & 0\\ k_{1} & -a_{2} & 0 & 0\\ 0 & 0 & -a_{3} & 0\\ 0 & 0 & k_{2} & -\mu_{v} \end{array}\right)}_{\left(DFE\right)}, \end{array}$$

where

*a*_1_ = *θ* + *k*_1_ + *μ*_*h*_, *a*_2_ = *β* + *μ*_*h*_, *a*_3_ = *k*_2_ + *μ*_*v*_.

The matrix say *K* = *FV*^−1^ obtained at disease-free equilibrium is referred as Next generation matrix for the given system. Each entry (*m*, *n*) of the Next generation matrix represents the expected number of secondary infections in compartment *m* caused by members of compartment *n*. The dominant nonnegative eigenvalue of the Next generation matrix is called Reproduction number, denoted by *R*_0_ [19]. The dominant eigen value of *ρ*(−*FV*^−1^) is:

$$\begin{array}{c} {\left[\frac{k_{1}k_{2}a^{2}b_{1}c_{1}\Gamma_{v}\mu_{h}}{\Gamma_{h}\left(k_{1}+\theta +\mu_{h}\right)\left(\beta +\mu_{h}\right)\left(k_{2}+\mu_{v}\right)\mu_{v}^{2}}\right]}^{\frac{1}{2}}. \end{array}$$

Which is the reproduction number

$$\begin{array}{c} R_{0}=\sqrt{\left[\frac{k_{1}k_{2}a^{2}b_{1}c_{1}\Gamma_{v}\mu_{h}}{\Gamma_{h}\left(k_{1}+\theta +\mu_{h}\right)\left(\beta +\mu_{h}\right)\left(k_{2}+\mu_{v}\right)\mu_{v}^{2}}\right]}. \end{array}$$

### 0.1 Biological Interpretation of Reproduction Number

In order to study the biological interpretation of *R*_0_ is given by

$$\begin{array}{c} R_{0}=\sqrt{\left[\frac{\left(ab_{1}\right)\left(ac_{1}\right)k_{2}k_{1}\Gamma_{v}\mu_{h}}{\Gamma_{h}\left(k_{1}+\theta +\mu_{h}\right)\left(\beta +\mu_{h}\right)\left(k_{2}+\mu_{v}\right)\mu_{v}^{2}}\right]}. \end{array}$$

Here *a* is the biting rate of sand fly, *b*_1_ is the transmission probability of *Cl* strain to human from sand fly and *c*_1_ is the transmission probability of *Cl* strain to sand fly from human.

If the human is susceptible and the sand fly is infected with *Cl*. The biting of human by sand fly, would result in the transmission of *Cl* stains to the human. The direction of transmission is denoted by the term *ab*_1_ in the *R*_0_. If the sand fly is not infected and the human is infected with *Cl*, then clearly *ac*_1_ is rightly indicating the secondary infections to sand fly from human. So *R*_0_ represents the transmission of *Cl* strains between human and sand fly. Thus *R*_0_ is biologically meaningful. Furthermore, we have emphasized *ac*_1_ and *ab*_1_, because we are interested in the direction of transmission. The rest of parameters used in *R*_0_ are concerned only with the magnitude and some others behavior in *R*_0_.

### Sensitivity Analysis of *R*_0_

The normalized forward sensitivity index of a variable to a parameter is the ratio of the relative change in the variable to the relative change in the parameter. If the variable is a differentiable function of the parameter, the sensitivity index is then defined using partial derivatives [23].

**Definition** The normalized forward sensitivity index of a variable *u* that depends differentiability on a parameter *p* is defined as:

$$\begin{array}{c} {ϒ}_{p}^{u}=\frac{\partial u}{\partial p}\frac{p}{u}. \end{array}$$

To reduce the rate of disease transmission, we need to know the importance of different factors involved in its transmission. Initial disease transmission depends on reproduction number *R*_0_. So we investigate the sensitivity indices of the reproduction number *R*_0_, relative to the parameters involved. The sensitivity indices of the reproduction number *R*_0_ is given in Table 1. These indices allow us to measure the relative change in *R*_0_ with the change in a parameter. Using these indices, we find the parameters that highly effect *R*_0_, and need to be targeted by intervention strategies.

**Table 1:** The sensitivity indices of the reproduction number *R*_0_.

Parameter	Sensitivity index	Index at parameters value
*a*	+1	+1
*β*	$\frac{-0.5\beta }{\beta +\mu_{h}}$	-0.4965
Γ_*v*_	+0.5	+0.5
Γ_*h*_	-0.5	-0.5
*b*_1_	0.5	+0.5
*c*_1_	0.5	+0.5
*k*_1_	$\frac{0.5(\Theta +\mu_{h})}{\left(k_{1}+\Theta +\mu_{h}\right)}$	+0.2345
*k*_2_	$\frac{0.5\mu_{h}}{k_{2}+\mu_{h}}$	+0.00009
*μ*_*v*_	$\frac{-0.5(\mu_{v}+2k_{2})}{k_{2}+\mu_{v}}$	-0.7571
*μ*_*h*_	$\frac{0.5(\beta k_{1}+\beta \Theta -\mu_{h}^{2})}{\left(\beta +\mu_{h}\right)\left(k_{1}+\Theta +\mu_{h}\right)}$	+0.4958

## Optimal Control Strategies

In this section, we use optimal control techniques to develop control strategies. In order to do this, we focus to control the transmission of infection like sand fly biting rate *a* has got highest sensitivity index 1. This means that decreases in biting rate by 10% would decrease *R*_0_ by 10%. The second highest index −0.7571 is that of sand fly’s mortality rate *μ*_*v*_. That is increasing *μ*_*v*_ by 10% will decrease *R*_0_ by 7.5%. The parameters Γ_*v*_, *b*_1_ and *c*_1_ have got sensitivity index of 0.5. By decreasing these parameters 10%, causes collective decrease of 15% in *R*_0_. Also Γ_*h*_ have got sensitivity index of -0.5. It is also to be noted that increase in human’s birth rate causes decrease in *R*_0_. The absolute sensitivity index of both treatment rate of human *β* and death rate of human *μ*_*h*_ is 0.49. So increasing the treatment rate of human by 10 percent will decrease *R*_0_ by 4.9%. Moreover, increase in treatment rate of human will cause decrease in human’s death rate, which will reduce *R*_0_.

Since the effect of all these parameters is coupled with three key parameters sand fly biting rate, treatment rate of infectious human and the mortality rate of sand fly. So instead of addressing all the parameters, we address the three key parameters; sand fly biting rate *a*, treatment rate of infectious human class and the mortality rate of sand flies which are main cause of transmission. Increase or decrease in these key parameters causes change in the rest of the parameters in the form of increase or decrease. For example decrease in sand fly biting rate *a* means decrease in the contact rate of human and sand fly. This creates difficulties for female sand fly to have human blood, which it needs for laying eggs. Consequently decrease occurs in sand fly birth rate Γ_*v*_. While decrease in contact rate of sand fly and human, reduce the chances of sand fly to catch infection from human or to transmit infection to human. This will reduce the transmission probability *b*_1_ and *c*_1_ of *ACL* between humans and sand flies.

In order to develop optimal control strategies, we introduce the following three optimal control variables:

- The control variable *u*_1_ represents the use of insecticide-treated bed nets and the use of sand fly’s repulsive lotions and electronic devices, to reduce sand fly biting rate.
- The control variable *u*_2_ shows the use of effective medicines for the treatment of infectious humans.
- The control variable *u*_3_ represents different measures like residual spraying of dwellings and animal shelters to kill sand flies at all stages.

By using these control variables our control problem becomes

$$\begin{array}{c} \left\{\begin{array}{c} \dot{S_{h}}\left(t\right)=\Gamma_{h}-\frac{a\left(1-u_{1}\right)b_{1}I_{v}\left(t\right)S_{h}\left(t\right)}{Nh(t)}-\mu_{h}S_{h}\left(t\right),\\ \dot{E_{h}}\left(t\right)=\frac{a\left(1-u_{1}\left(t\right)\right)b_{1}I_{v}\left(t\right)S_{h}\left(t\right)}{Nh(t)}-\left(k_{1}+\theta +\mu_{h}\right)E_{h}\left(t\right),\\ \dot{I_{h}}\left(t\right)=k_{1}E_{h}\left(t\right)-\left(u_{2}\left(t\right)+\beta +\mu_{h}\right)I_{h}\left(t\right),\\ \dot{R_{h}}\left(t\right)=\theta E_{h}\left(t\right)+\left(u_{2}\left(t\right)+\beta \right)I_{h}\left(t\right)-\mu_{h}R_{h}\left(t\right),\\ \dot{S_{v}}\left(t\right)=\Gamma_{v}-\frac{a\left(1-u_{1}\left(t\right)\right)c_{1}I_{v}\left(t\right)S_{v}\left(t\right)}{Nh(t)}-\left(u_{3}\left(t\right)+\mu_{v}\right)S_{v}\left(t\right),\\ \dot{E_{v}}\left(t\right)=\frac{a\left(1-u_{1}\left(t\right)\right)c_{1}I_{h}\left(t\right)S_{v}\left(t\right)}{Nh(t)}-\left(u_{3}\left(t\right)+\mu_{v}+k_{2}\right)E_{v}\left(t\right),\\ \dot{I_{v}}\left(t\right)=k_{2}E_{v}\left(t\right)-\left(u_{3}+\mu_{v}\right)I_{v}\left(t\right). \end{array}\right. \end{array}$$ (4)

The goal of our optimal control strategies is to minimize the infectious and exposed human population, the vector population, sand fly biting rate and the cost of implementing the control by using possible minimal control variables *u*_1_(*t*), *u*_2_(*t*) and *u*_3_(*t*). In order to do this, we using the bounded Lebesgue measurable control to construct the objective functional is given by

$$\begin{array}{c} J\left(u_{1},u_{2},u_{3}\right)=\int_{0}^{T}\left(g_{1}E_{h}\left(t\right)+g_{2}I_{h}\left(t\right)+g_{3}\left(S_{v}\left(t\right)+E_{v}\left(t\right)+I_{v}\left(t\right)\right)+\frac{1}{2}\left(d_{1}u_{1}^{2}\left(t\right)+d_{2}u_{2}^{2}\left(t\right)+d_{3}u_{3}^{2}\left(t\right)\right)\right)dt \end{array}$$ (5)

subject to the system (4).

In the objective functional, *g*_1_, *g*_2_ and *g*_3_ represent the weight constants of the exposed, infectious human and of vector population, respectively. *d*_1_, *d*_2_ and *d*_3_ are weight constants of human self protection, human treatment and vector control, respectively. The terms $\left(1/2\right)d_{1}u_{1}^{2}\left(t\right),\left(1/2\right)d_{2}u_{2}^{2}\left(t\right)$ and $\left(1/2\right)d_{3}u_{3}^{2}\left(t\right)$ described the cost of disease interventions. The cost associated with the first control strategy *u*_1_ comes from the cost of sand fly repellent lotions, electric mats and mosquito bed nets. The cost associated with the second control strategy *u*_2_(*t*), is the cost of expensive medication of human class. The cost associated with the third control strategy *u*_3_(*t*), could arise from applying different types chemical pesticides, to kill sand fly at any stage of its life. We have assumed the costs as proportional to the square of the corresponding control function. We aim to find control functions so that:

$$\begin{array}{c} J\left(u_{1}^{*},u_{2}^{*},u_{3}^{*}\right)=min\left\{J\left(u_{1},u_{2},u_{3}\right),\left(u_{1},u_{2},u_{3}\right)\in D\right\} \end{array}$$

subject to the system (4). The control set *D* is defined as:

$$\begin{array}{c} D=\{\left(u_{1},u_{2},u_{3}\right)|u_{i}\left(t\right)\;is\;Lebesgue\;measurable\;on\;\left[0,1\right],0\leq u_{i}\left(t\right)<1,i=1,2,3\}. \end{array}$$

### Existence of the Control Problem

For existence of the control problem, we consider the control system (4) with initial conditions at *t* = 0. In case of bounded Lebesgue measurable controls and non-negative initial conditions, there exist non-negative bounded solution of the state system [14, 24]. For optimal solution of the system, first we find the Lagrangian and Hamiltonian. We define the Lagrangian of the control problem Eq (4) is given by

$$\begin{array}{c} L\left(t\right)=g_{1}E_{h}\left(t\right)+g_{2}I_{h}\left(t\right)+g_{3}N_{v}\left(t\right)+\frac{1}{2}\left(d_{1}u_{1}^{2}\left(t\right)+d_{2}u_{2}^{2}\left(t\right)+d_{3}u_{3}^{2}\left(t\right)\right), \end{array}$$ (6)

where *N*_*v*_(*t*) = *S*_*v*_(*t*) + *E*_*v*_(*t*) + *I*_*v*_(*t*).

We define the Hamiltonian H, to find the minimal value of the Lagrangian, as follow:

$$\begin{array}{c} \begin{array}{ccc} H(t) & = & L\left(t\right)+\lambda_{1}\left(t\right)\frac{dS_{h}\left(t\right)}{dt}+\lambda_{2}\left(t\right)\frac{dE_{h}\left(t\right)}{dt}+\lambda_{3}\left(t\right)\frac{dI_{h}\left(t\right)}{dt}\\ & & +\lambda_{4}\left(t\right)\frac{dR_{h}\left(t\right)}{dt}+\lambda_{5}\left(t\right)\frac{dS_{v}\left(t\right)}{dt}+\lambda_{6}\left(t\right)\frac{dE_{v}\left(t\right)}{dt}+\lambda_{7}\left(t\right)\frac{dI_{v}\left(t\right)}{dt}. \end{array} \end{array}$$ (7)

**Theorem** There exists an optimal control $u^{*}=\left(u_{1}^{*},u_{2}^{*},u_{3}^{*}\right)\in D$ such that *J*(*u*_1_, *u*_2_, *u*_3_) = *min*_(*u*_1_, *u*_2_, *u*_3_)∈*D*_ *J*(*u*_1_, *u*_2_, *u*_3_) subject to the system (4) and initial conditions at *t* = 0.

**Proof** The control variables and the state variables are nonnegative. So we use the result in [14, 25], for the existence of an optimal control. The necessary convexity of the objective functional in *u*_1_, *u*_2_ *and* *u*_3_ are satisfied here. Also by definition, the set of the control variables (*u*_1_, *u*_2_, *u*_3_) ∈ *D*, is convex and closed. The compactness which we need for the existence of the optimal control is confirmed by boundedness of the optimal system. Also, the integrand in the functional Eq (5), $g_{1}E_{h}+g_{2}I_{h}+g_{3}N_{v}+\frac{1}{2}\left(d_{1}u_{1}^{2}+d_{2}u_{2}^{2}+d_{3}u_{3}^{2}\right)$ is convex on the control set D. We can find a constant *η* > 1 and positive numbers *ξ*_1_ and *ξ*_2_ such that $J\left(u_{1},u_{2},u_{3}\right)\geq \xi_{1}\left(\right|u_{1}{|}^{2},|u_{2}{|}^{2},|u_{3}{{|}^{2})}^{\frac{\eta }{2}}-\xi_{2}$, as the state variables are bounded. Which completes the proof of existence of an optimal control.

For optimal solution, we apply Pontryagin’s Maximum Principle [26] as follow. If (*x*(*t*), *u*(*t*)) is an optimal solution of an optimal control problem, then there exists a non trivial vector function *λ*(*t*) = (*λ*_1_(*t*), *λ*_2_(*t*), …, *λ*_*n*_(*t*)) satisfying the following:

$$\frac{dx(t)}{dt}=\frac{\partial H(t,x,u,\lambda )}{\partial \lambda ,}$$

$$0=\frac{\partial H(t,x,u,\lambda )}{\partial u,}$$

$$\lambda^{\prime }\left(t\right)=-\frac{\partial H(t,x,u,\lambda )}{\partial x.}$$

We apply the necessary conditions to the Hamiltonian H in the system (7).

**Theorem** let $S_{h}^{*},E_{h}^{*},I_{h}^{*},R_{h}^{*},S_{v}^{*},E_{v}^{*}\;and\;I_{v}^{*}$ be optimal state solutions with associated optimal control variables $\left(u_{1}^{*},u_{2}^{*},u_{3}^{*}\right)$ for the optimal control problem Eqs (4) and (5). Then there exist adjoint variables *λ*_*i*_ *for* *i* = 1, 2, …, 7 satisfying

$$\{\begin{array}{ccccc} \frac{d\lambda_{1}(t)}{dt} & = & \frac{\left(\lambda_{1}(t)-\lambda_{2}(t))ab_{1}I_{v}^{*}(E_{h}^{*}+I_{h}^{*}+R_{h}^{*}\right)}{N_{h}^{2}}+\frac{(\lambda_{6}(t)-\lambda_{5}(t))ac_{1}I_{h}^{*}S_{v}^{*}}{N_{h}^{2}}+\lambda_{1}(t)\mu_{h}, & & \\ \frac{d\lambda_{2}(t)}{dt} & = & \frac{(\lambda_{2}(t)-\lambda_{1}(t))ab_{1}I_{v}^{*}S_{h}^{*}}{N_{h}^{2}}+\frac{(\lambda_{6}(t)-\lambda_{5}(t))ac_{1}(1-u_{1})I_{h}^{*}S_{v}^{*}}{N_{h}^{2}}+(\lambda_{2}(t)-\lambda_{3}(t))k_{1} & +(\lambda_{2}(t)-\lambda_{4}(t))\Theta +\lambda_{2}(t)\mu_{h}-g_{1}, & \\ \frac{d\lambda_{3}(t)}{dt} & = & \frac{\left(\lambda_{2}(t)-\lambda_{1}(t))ab_{1}I_{v}^{*}S_{h}^{*}(1-u_{1}\right)}{N_{h}^{2}}+\frac{\left(\lambda_{6}(t)-\lambda_{5}(t))ac_{1}(1-u_{1})S_{v}^{*}(S_{h}^{*}+E_{h}^{*}+R_{h}^{*}\right)}{N_{h}^{2}} & +(\lambda_{3}-\lambda_{4}(t))(u_{2}+\beta )+\lambda_{3}(t)\mu_{h}-g_{2}, & \\ \frac{d\lambda_{4}(t)}{dt} & = & \frac{\left(\lambda_{2}(t)-\lambda_{1}(t))ab_{1}I_{v}^{*}S_{h}^{*}(1-u_{1}\right)}{N_{h}^{2}}+\frac{(\lambda_{6}(t)-\lambda_{5}(t))ac_{1}I_{h}^{*}S_{v}^{*}}{N_{h}^{2}}+\lambda_{4}(t)\mu_{h}, & & \\ \frac{d\lambda_{5}(t)}{dt} & = & \frac{\left(\lambda_{5}(t)-\lambda_{6}(t))ac_{1}I_{h}^{*}(1-u_{1}\right)}{N_{h}}+\lambda_{3}(t)(u_{3}+\mu_{v})-g_{3}, & & \\ \frac{d\lambda_{6}(t)}{dt} & = & (\lambda_{6}(t)-\lambda_{7}(t))k_{2}+\lambda_{6}(t)(u_{3}+\mu_{v})-g_{3}, & & \\ \frac{d\lambda_{7}(t)}{dt} & = & \frac{(\lambda_{1}(t)-\lambda_{2}(t))ab_{1}S_{h}^{*}}{N_{h}}+\lambda_{7}(t)(u_{3}+\mu_{v})-g_{3}. & & \end{array}$$ (8)

with transversality conditions (or boundary conditions)

$$\begin{array}{c} \lambda_{i}\left(t_{end}\right)=0\;for\;i=1,2,..,7. \end{array}$$ (9)

Furthermore, optimal controls $u_{1}^{*},u_{2}^{*}$ and $u_{3}^{*}$ are given by

$$\begin{array}{c} u_{1}^{*}\left(t\right)=max\left\{min\left\{\frac{a\left(\lambda_{2}-\lambda_{1}\right)b_{1}I_{v}^{*}S_{h}^{*}+\left(\lambda_{6}-\lambda_{5}\right)ac_{1}I_{h}^{*}S_{v}^{*}}{d_{1}N_{h}},1\right\},0\right\}, \end{array}$$ (10)

$$\begin{array}{c} u_{2}^{*}\left(t\right)=max\left\{min\left\{\frac{\left(\lambda_{3}-\lambda_{4}\right)I_{h}^{*}}{d_{2}},1\right\},0\right\}, \end{array}$$ (11)

$$\begin{array}{c} u_{3}^{*}\left(t\right)=max\left\{min\left\{\frac{\lambda_{5}S_{v}^{*}+\lambda_{6}E_{v}^{*}+\lambda_{7}I_{v}^{*}}{d_{3}},1\right\},0\right\}. \end{array}$$ (12)

**Proof**: To find adjoint equations and the transversality conditions, we use Eq (7). Differentiating the Hamiltonian *H* with respect to each state variables, we obtain the system (8). Solving the equations $\frac{\partial H}{\partial u_{1}}=0$, $\frac{\partial H}{\partial u_{2}}=0$ and $\frac{\partial H}{\partial u_{3}}=0$ on the interior of the control set and using the optimality conditions and the property of the control space D, we can derive Eqs (10)–(12). The optimal control and the state are found by solving the optimality system (5), the adjoint system $\frac{d\lambda_{i}}{dt}$, initial and boundary conditions (9), equations of the optimal control Eqs (10)–(12). Since the second derivatives of the Lagrangian with respect to *u*_1_, *u*_2_ and *u*_3_ are positive, so optimal problem is minimum at controls $u_{1}^{*}$, $u_{2}^{*}$, and $u_{3}^{*}$. By substituting the values of $u_{1}^{*}$, $u_{2}^{*}$, and $u_{3}^{*}$ in the control system (7), we get the following system

$$\begin{array}{c} \left\{\begin{array}{cc} \dot{S_{h}^{*}}\left(t\right) & =\Gamma_{h}-\frac{\left(1-max\left[min\left\{\frac{a\left(\lambda_{2}\left(t\right)-\lambda_{1}\left(t\right)\right)b_{1}I_{v}^{*}\left(t\right)S_{h}^{*}\left(t\right)+\left(\lambda_{6}\left(t\right)-\lambda_{5}\left(t\right)\right)c_{1}I_{h}^{*}\left(t\right)S_{v}^{*}\left(t\right))}{B_{1}N_{h}\left(t\right)},1\right\},0\right]\right)ab_{1}I_{v}^{*}S_{h}^{*}\left(t\right)}{Nh(t)}-\mu_{h}S_{h}^{*}\left(t\right),\\ \dot{E^{*}}\left(t\right) & =\frac{\left(1-max\left[min\left\{\frac{a\left(\lambda_{2}\left(t\right)-\lambda_{1}\left(t\right)\right)b_{1}I_{v}^{*}\left(t\right)S_{h}^{*}\left(t\right)+\left(\lambda_{6}\left(t\right)-\lambda_{5}\left(t\right)\right)c_{1}I_{h}^{*}\left(t\right)S_{v}^{*}\left(t\right))}{B_{1}N_{h}\left(t\right)},1\right\},0\right]\right)ab_{1}I_{v}^{*}\left(t\right)S_{h}^{*}\left(t\right)}{Nh(t)}-\left(k_{1}+\theta +\mu_{h}\right)E_{h}^{*}\left(t\right),\\ \dot{I^{*}}\left(t\right) & =k_{1}E^{*}-\left(max\left[min\left\{\frac{a(\lambda_{3}\left(t\right)-\lambda_{4}\left(t\right))I}{B_{2}},1\right\},0\right]\right)I^{*}-\left(\beta +\mu_{h}\right)I_{h}^{*}\left(t\right),\\ \dot{R^{*}}\left(t\right) & =\theta E^{*}+\left(max\left[min\left\{\frac{a\left(\lambda_{3}\left(t\right)-\lambda_{4}\left(t\right)\right)I_{h}^{*}\left(t\right)}{B_{2}},1\right\},0\right]\right)I_{h}^{*}\left(t\right)+\left(\beta \right)I_{h}^{*}\left(t\right)-\mu_{h}R_{h}^{*}\left(t\right),\\ \dot{S_{v}^{*}}\left(t\right) & =\Gamma_{v}-\frac{\left(1-max\left[min\left\{\frac{a\left(\lambda_{2}\left(t\right)-\lambda_{1}\left(t\right)\right)b_{1}I_{v}^{*}S_{h}^{*}+\left(\lambda_{6}\left(t\right)-\lambda_{5}\left(t\right)\right)c_{1}I_{h}^{*}\left(t\right)S_{v}^{*}\left(t\right))}{B_{1}N_{h}\left(t\right)},1\right\},0\right]\right)ac_{1}I_{h}^{*}\left(t\right)S_{v}^{*}\left(t\right)}{Nh(t)}\\ & -max\left\{min\left\{\frac{\lambda_{5}\left(t\right)S_{v}^{*}\left(t\right)+\lambda_{6}\left(t\right)E_{v}^{*}\left(t\right)+\lambda_{7}\left(t\right)I_{v}^{*}\left(t\right)}{B_{3}},1\right\},0\right\}S_{v}^{*}\left(t\right)-\left(\mu_{v}\right)S_{v}^{*}\left(t\right),\\ \dot{E_{v}^{*}}\left(t\right) & =-\frac{\left(1-max\left[min\left\{\frac{a\left(\lambda_{2}\left(t\right)-\lambda_{1}\left(t\right)\right)b_{1}I_{v}^{*}S_{h}^{*}+\left(\lambda_{6}\left(t\right)-\lambda_{5}\left(t\right)\right)c_{1}I_{h}^{*}\left(t\right)S_{v}^{*}\left(t\right))}{B_{1}N_{h}\left(t\right)},1\right\},0\right]\right)ac_{1}I_{h}^{*}\left(t\right)S_{v}^{*}\left(t\right)}{Nh(t)}\\ & -max\left\{min\left\{\frac{\lambda_{5}\left(t\right)S_{v}^{*}+\lambda_{6}\left(t\right)E_{v}^{*}\left(t\right)+\lambda_{7}\left(t\right)I_{v}^{*}\left(t\right)}{B_{3}},1\right\},0\right\}E_{v}^{*}\left(t\right)-\left(\mu_{v}+k_{2}\right)E_{v}^{*}\left(t\right),\\ \dot{I_{v}^{*}}\left(t\right) & =k_{2}E_{v}^{*}\left(t\right)-max\left\{min\left\{\frac{\lambda_{5}\left(t\right)S_{v}^{*}\left(t\right)+\lambda_{6}\left(t\right)E_{v}^{*}\left(t\right)+\lambda_{7}\left(t\right)I_{v}^{*}\left(t\right)}{B_{3}},1\right\},0\right\}I_{h}^{*}\left(t\right)-\left(\mu_{v}\right)I_{v}^{*}\left(t\right). \end{array}\right. \end{array}$$ (13)

with

$$\begin{array}{c} \begin{array}{ccc} H^{*}\left(t\right) & = & A_{1}E_{h}^{*}\left(t\right)+A_{2}I_{h}^{*}\left(t\right)+A_{3}N_{v}^{*}\left(t\right)+\frac{1}{2}(d_{1}{\left(u_{1}^{*}\left(t\right)\right)}^{2}+d_{2}{\left(u_{2}^{*}{\left(t\right))}^{2}+d_{3}{\left(u_{3}^{*}\left(t\right)\right)}^{2}\right)}^{2}+\lambda_{1}\left(t\right)\frac{dS_{h}^{*}\left(t\right)}{dt}\\ & & +\lambda_{2}\left(t\right)\frac{dE_{h}^{*}}{dt}+\lambda_{3}\left(t\right)\frac{dI_{h}^{*}\left(t\right)}{dt}+\lambda_{4}\left(t\right)\frac{dR_{h}^{*}\left(t\right)}{dt}+\lambda_{5}\left(t\right)\frac{dS_{v}^{*}\left(t\right)}{dt}+\lambda_{6}\left(t\right)\frac{dE_{v}^{*}\left(t\right)}{dt}+\lambda_{7}\left(t\right)\frac{dI_{v}^{*}\left(t\right)}{dt}. \end{array} \end{array}$$ (14)

## Numerical Simulations

In this section, we present numerical simulation. For numerical simulation, we use fourth order Runge-Kutta forward method. First, we use fourth order Runge-Kutta forward in time to solve the system (4), with estimated controls, over the simulated time. We then solve the system (8) with the help of backward method and the condition (9), utilizing the current iteration of the state equations. We apprise the controls *u*_1_, *u*_2_ and *u*_3_ through convex combination of both the previous iteration’s controls and the value of the characterization of Eqs (10)–(12). By keeping continue in this way until the values of unknowns at the consecutive iterations are too closed. For more detail see [27]. The value of weight constants used in the objective functional are *g*_1_ = 0.081, *g*_2_ = 0.01, *g*_3_ = 0.04, *d*_1_ = 0.08, *d*_2_ = 0.01 and *d*_3_ = 0.04. We used initial value of the state variables such as *S*_*h*_(0) = 100, *E*(0) = 20, *I*(0) = 20, *R*(0) = 10, *S*_*v*_(0) = 1000, *E*_*v*_(0) = 20 and *I*_*v*_(0) = 30.

Since the proposed model is composed of coupled system of nonlinear differential equations. Therefore applying control strategy to any targeted class say *x*_*t*_ of the model, will also effect the rest of the classes say *x*_*r*_, in the model. In other words *x*_*t*_ is directly effected and *x*_*r*_ are indirectly effected by the applied strategy.

## Discussion and Conclusion

**Fig 2:**
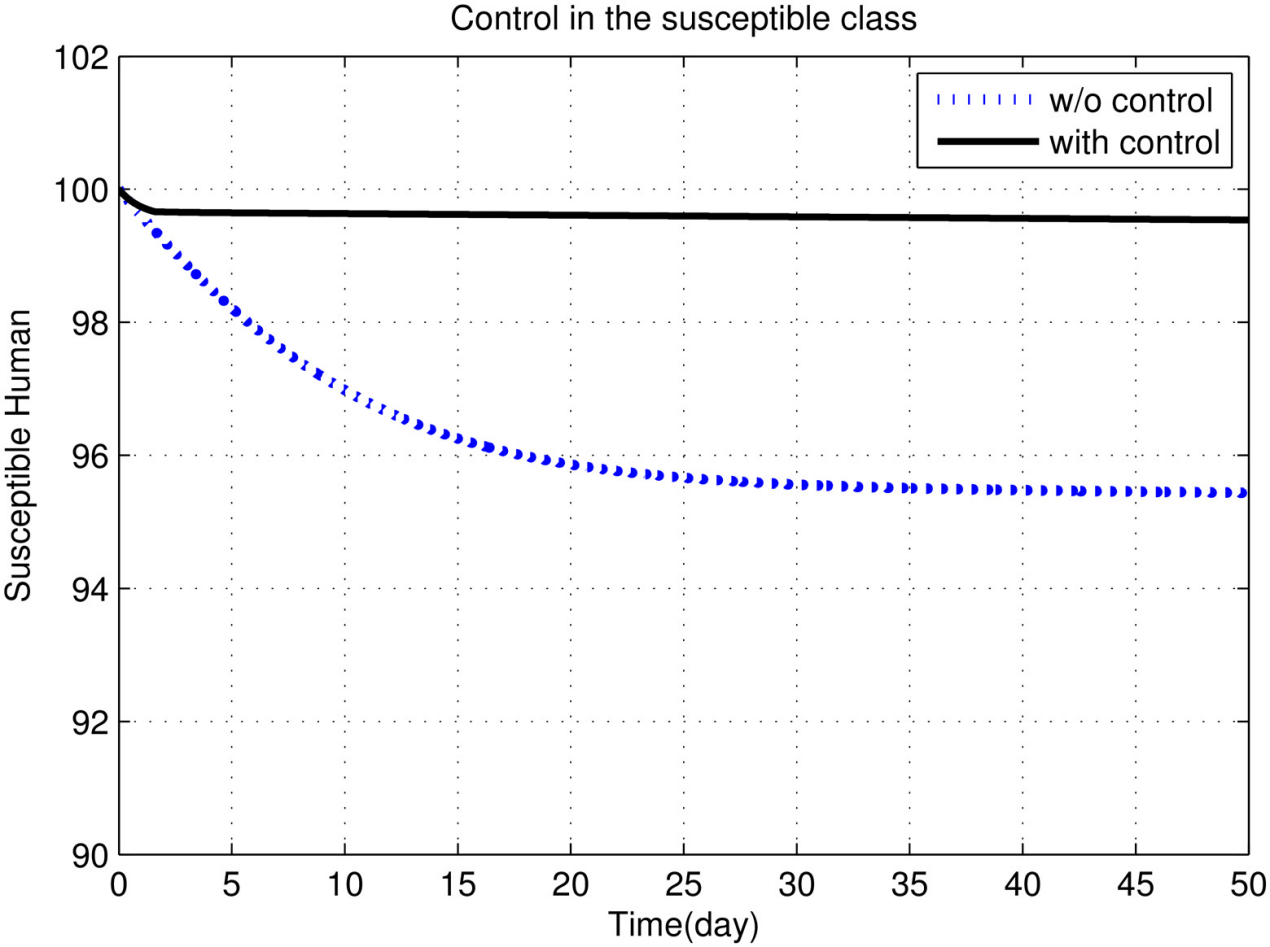
The graph represents the susceptible humans with and without control. The number of susceptible individuals approach to a small number due to optimal control.

**Fig 3:**
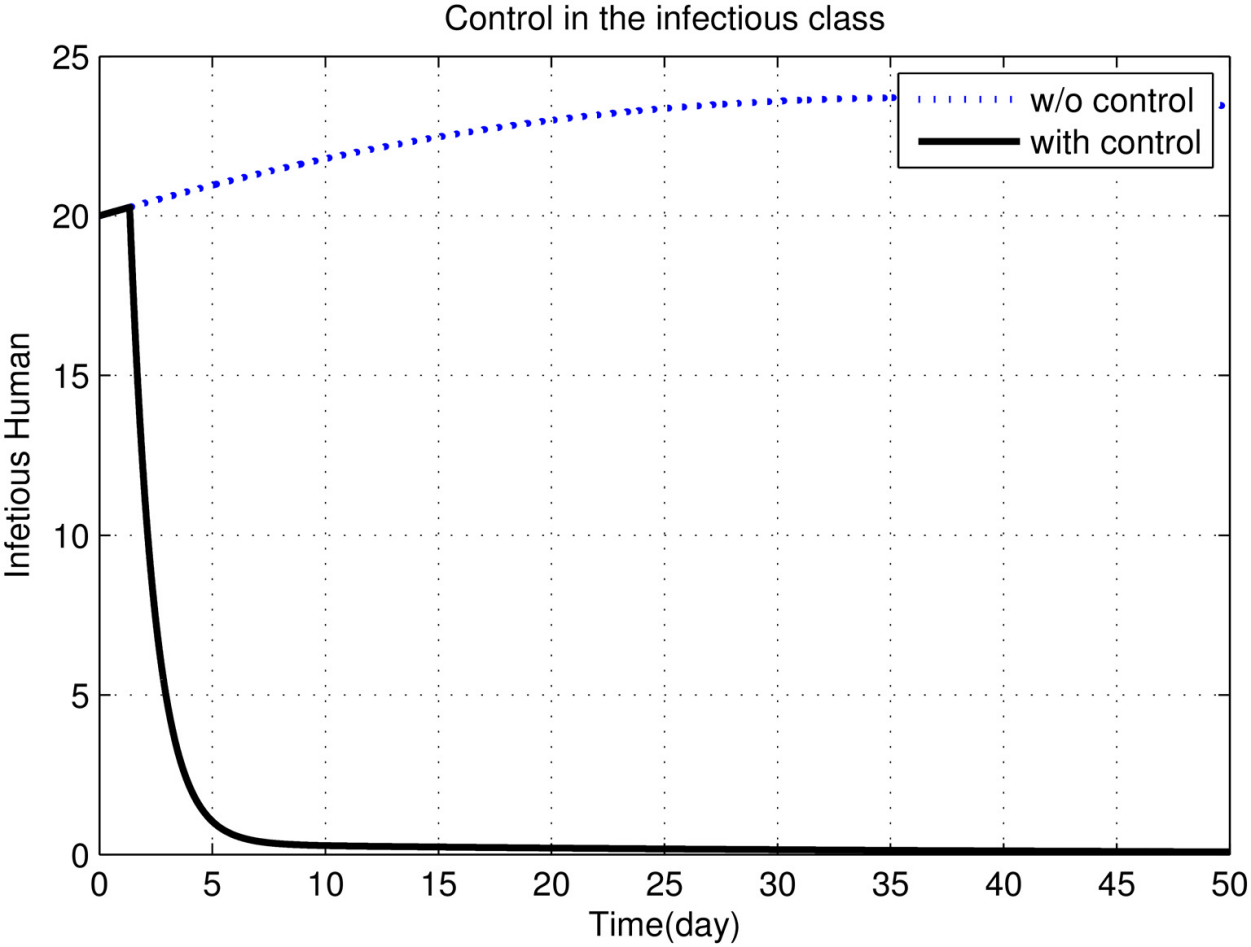
The graph represents the infectious humans with and without control. The number of infectious individuals approach to a small number due to optimal control.

**Fig 4:**
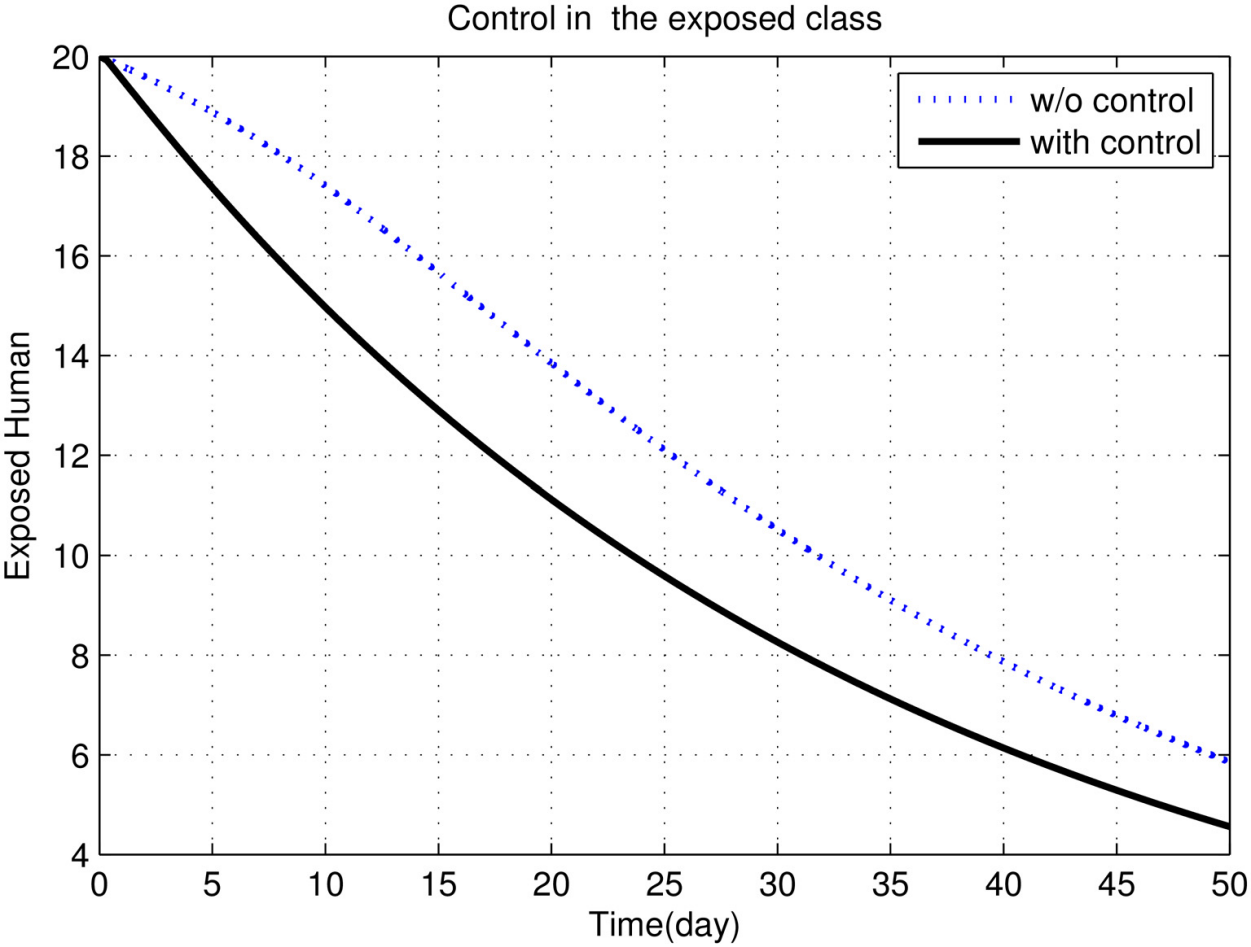
The graph shows the exposed humans with and without control.

**Fig 5:**
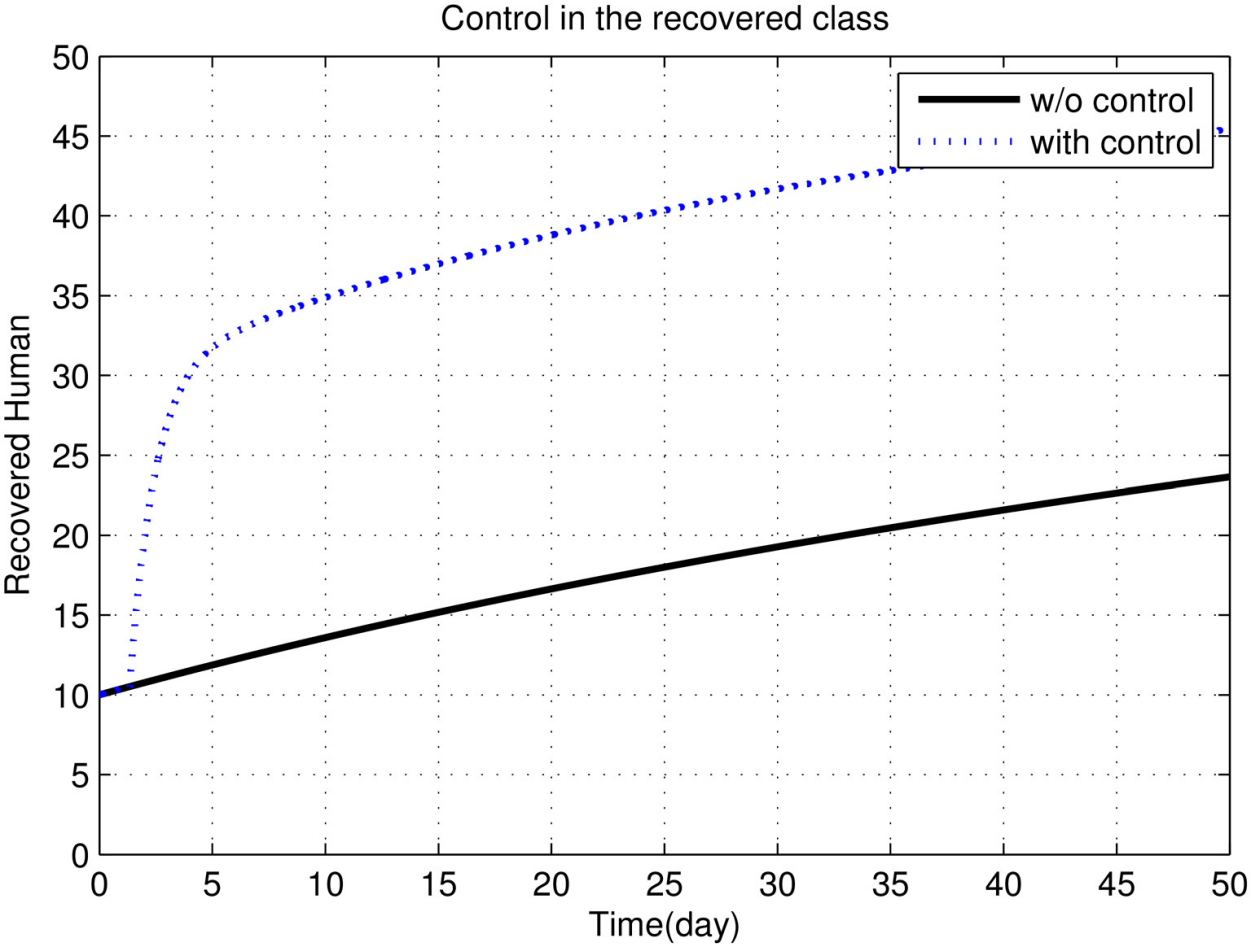
The graph represents the recovered humans with and without control.

**Fig 6:**
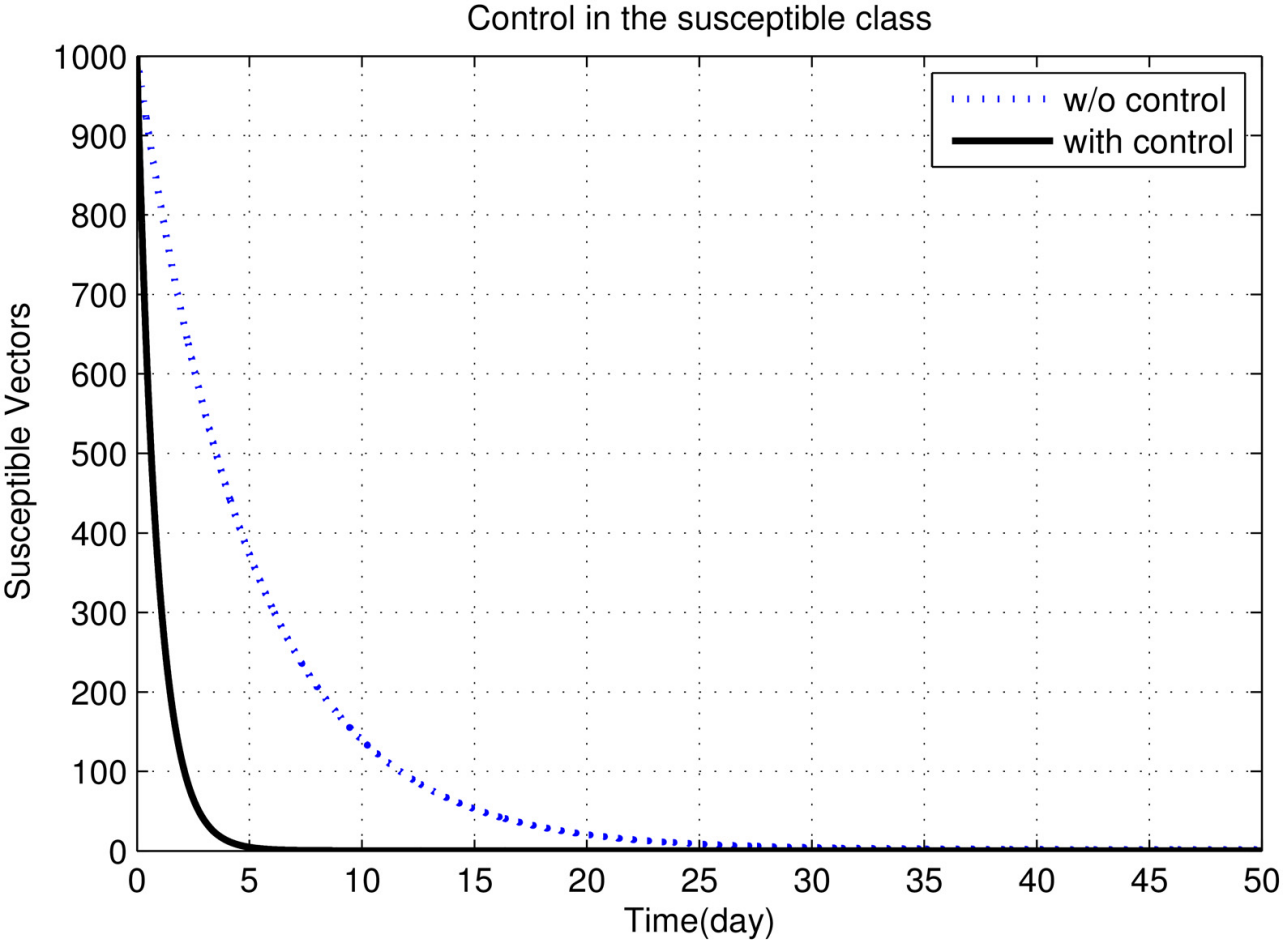
The graph represents the susceptible vectors with and without control. The number susceptible sand flies approach to a small number, due to optimal control.

**Fig 7:**
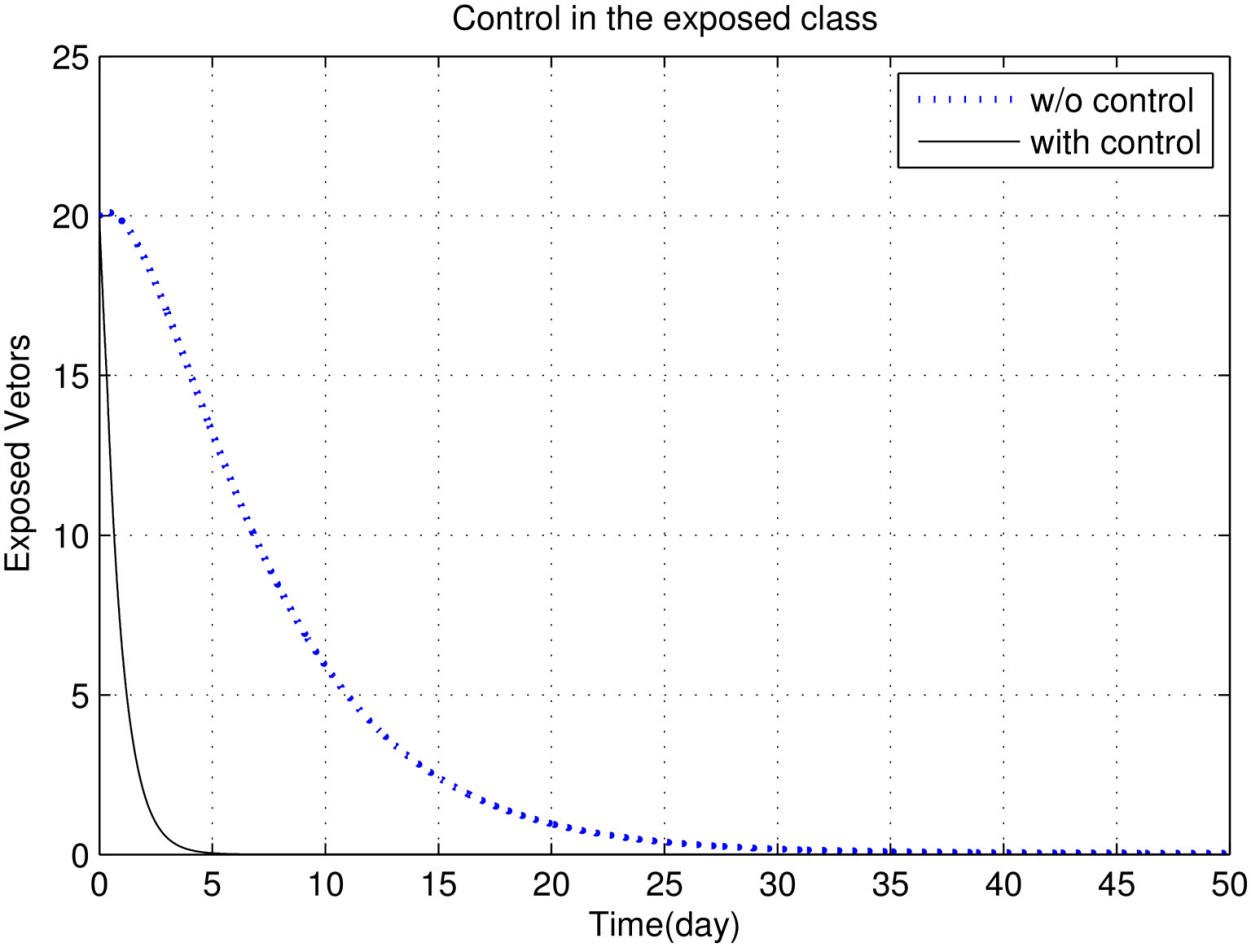
The graph represents the exposed vectors with and without control. The number of exposed sand flies approach to a small number, due to optimal control.

**Fig 8:**
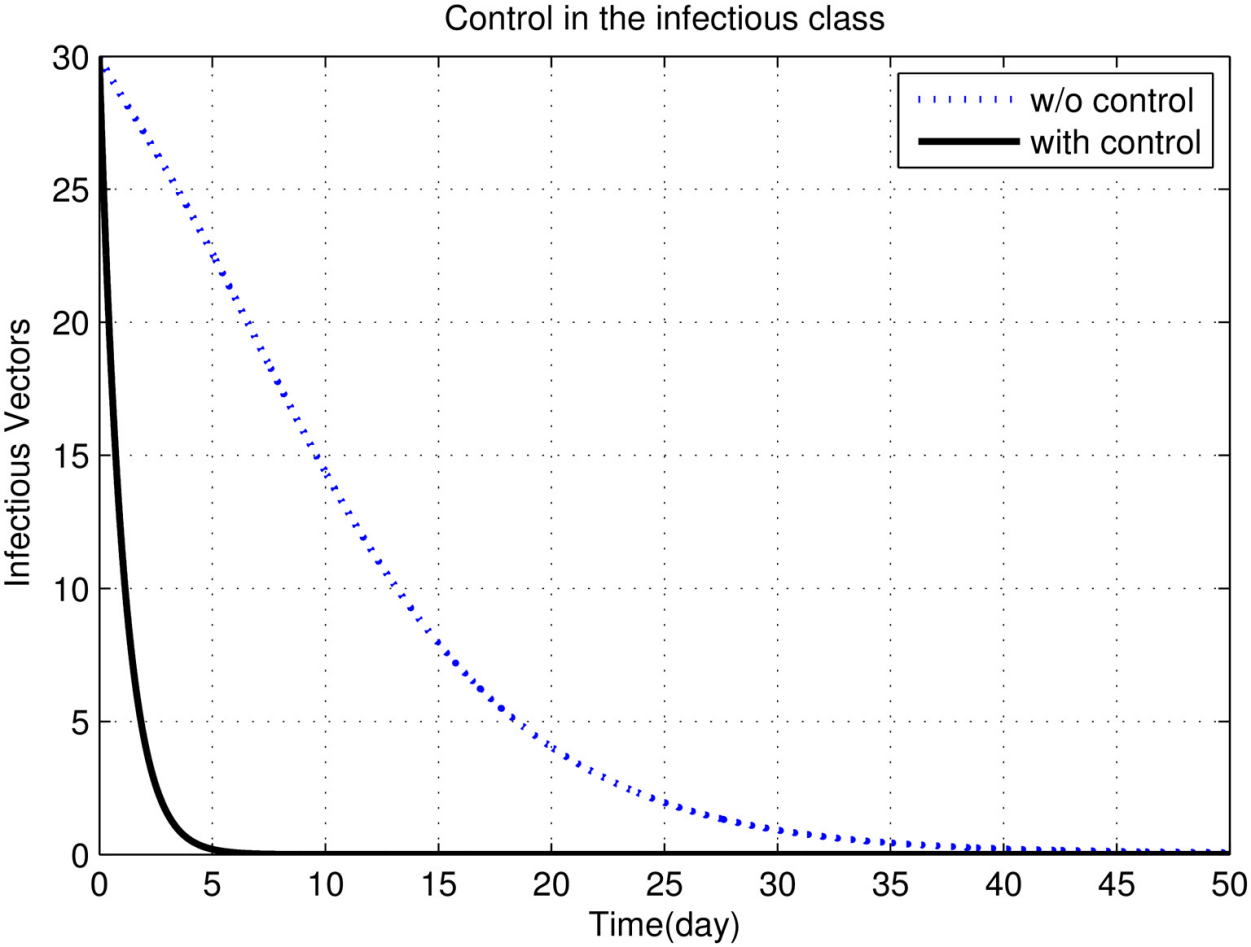
The graph shows infected vectors with and without control. The number of infected sand flies approach to a small number, due to optimal control.

First, we discuss results of numerical simulations. The control variable *u*_1_ reduces sand fly biting rate *a*, which causes decrease in the contact rate of humans and sand flies and consequently decrease in exposed and infectious classes of human as shown in Figs 2 to 8. The graph in Fig 2 represents the susceptible humans with and without control. The number of susceptible individuals approach to a small number due to optimal control. In Fig 3 The number of infectious individuals approach to a small number due to optimal control. The exposed and recovered humans with and without control are represented in Figs 4 and 5, respectively. The graph in Fig 6 shown the susceptible vectors with and without control. The number susceptible sand flies approach to a small number, due to optimal control. Figs 7 and 8 shown the graph the exposed and infected vectors with and without control, respectively. The number of exposed sand flies approach to a small number, due to optimal control. The control variable *u*_1_ indirectly effect the infectious class of human, where the control variable *u*_2_, directly effect the infectious class of humans. This is the reason that reduction in the infectious class is rapid as compared the reduction in exposed class of human. Fig 3 shows that for the initial period of few days, an increase occur in the infectious class of human. There are two reasons for this increase:

- Applying the control *u*_1_, minimize the number of new entries to the exposed human class. But those humans, whom are already exposed, get infectious after completing incubation period, which causes increase in the infectious class of human for initial few days.
- The medication *u*_2_, takes some time to show its effect, because the infectious human does not recover immediate after the use of medicine.

Recovered human class depends on both infectious and exposed human classes. Therefore an increase is observed in the recovered human class, with the decrease in both infectious and exposed human classes, as shown Figs 4 and 5.

**Fig 9:**
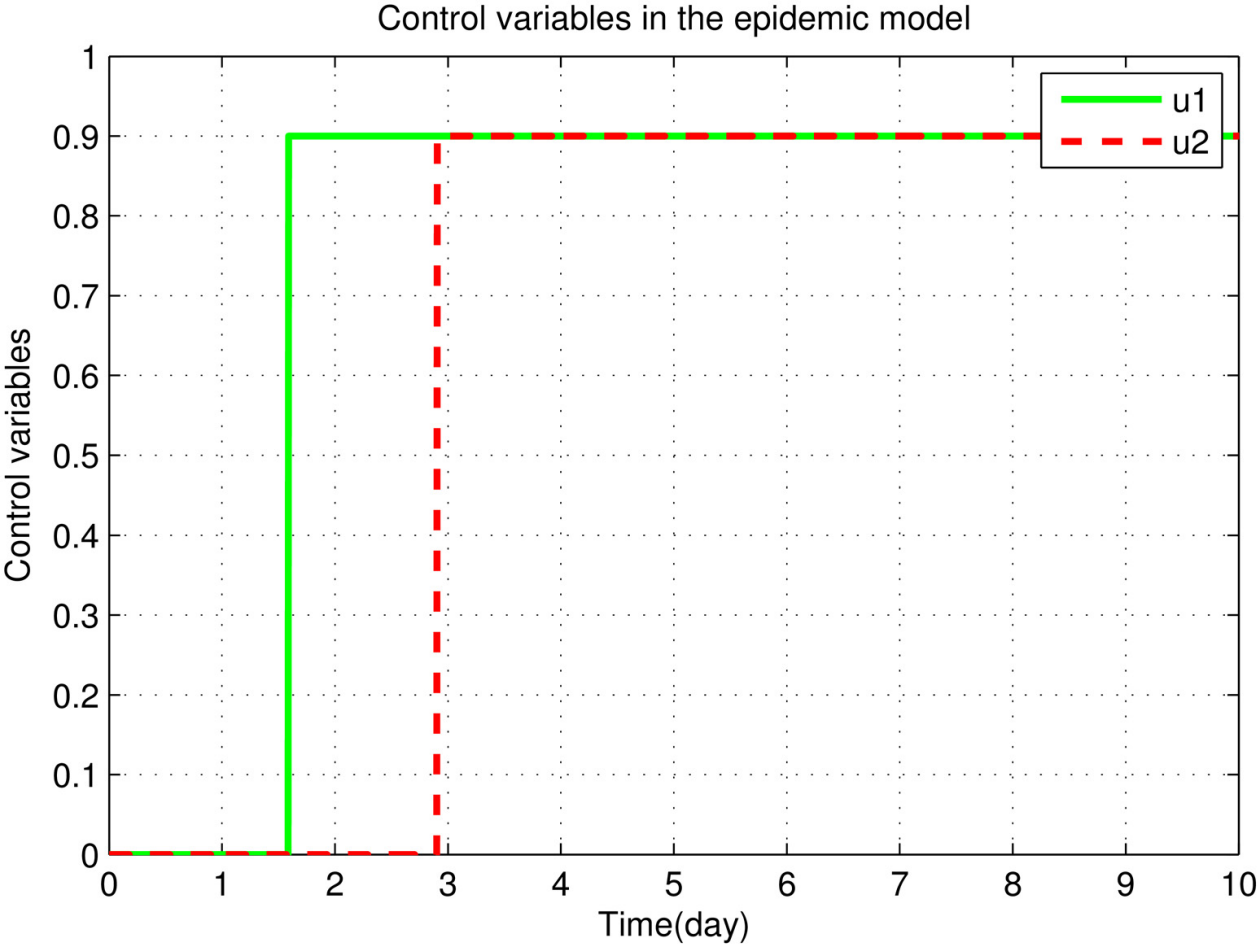
The graph shows the control variables *u*_1_ and *u*_2_.

To control the spread of vector population we apply control variable *u*_3_. Figs 7 and 8 show that reduction in vector population is very rapidly. There are three reasons for this rapid decrease:

1. The control variable *u*_1_ minimize the biting rate of sand fly biting rate, which effects vector population in two different ways:
  - Female sand fly needs human blood before laying eggs. Therefore, reduction in sand fly biting rate causes decrease in the birth rate of sand fly and consequently a decrease in the number of susceptible vectors.
  - The control *u*_1_, reduces the contact rate of human and sand fly. Therefore the chances of susceptible vector to interact the infectious human decreases. Hence the rate of disease transmission from human to sand fly decreases, which causes reduction in the exposed and infectious classes of vectors.
2. The control *u*_2_ minimize the number of infectious humans, which reduces the chances of susceptible sand fly to catch infection from infectious human. This causes decrease in the number of exposed and infectious vectors represented in Fig 9.
3. The control variable *u*_3_, directly effects the vector population. This control tries to kill sand flies in all compartments of the vector population.

Fig 9 shows that the efficiency of the control variable *u*_1_ is lowest for initial few days and remains maximum rest of the time. Similarly the efficiency of the control variable *u*_2_ is minimum for initial few days and remains maximum rest of the time. The reason is that both the strategies; medication of infectious human and the use of sand fly repulsive lotions, take time to show effect.

**Fig 10:**
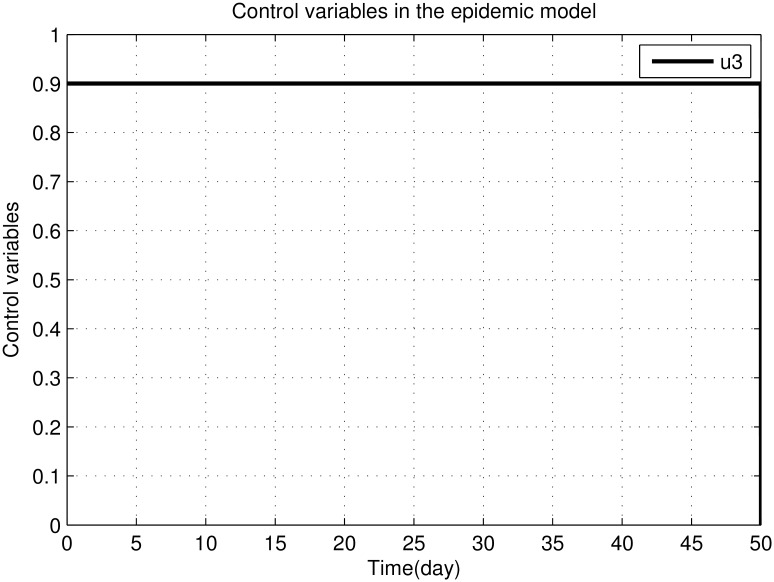
The graph shows the control variable *u*_3_.

The efficiency of the control variable *u*_3_ is maximum all the times represented in Fig 10. Because the strategy is due to kill the sand flies with direct source, with immediate effect.

We analyzed the mathematical model of *Anthroponotic Cutaneous Leishmania* using sensitivity indices of the reproduction number *R*_0_. With help of these sensitivity indices we found the relative importance of the role of different parameters, in the transmission of *ACL*. We addressed three key parameters; sand fly biting rate, mortality rate of sand flies and healing (recovery) rate of infectious humans. For this we introduced three control variables (strategies), in the optimal control problem, each for reducing sand fly biting rate, increasing mortality rate of sand fly and increasing recovery rate of humans. Our control strategies caused decrease in initial transmission rate *R*_0_. Since the control strategies are always effected by economics constraints, therefore, we have taken into account the constraints imposed by limited resources, in our objective functional. The control system was analyzed through Pontryagin’s Maximum Principle, to determine the necessary conditions for existence of optimal control problem. The results obtained from numerical simulations show that the control strategies are very effective, if implemented, on the same time, in the same area. And hence it is concluded that proposed optimal control works well in eradication and blocking further transmission of the *Anthroponotic Cutaneous Leishmania*.

We would like to extend this work by incorporating different biological behaviour in the future for instance:

1. The impact of temperature on the birth rate of sand flies and hence the transmission rate of Leishmaniasis, and to design a time and temperature dependent mathematical model. Since temperature of different regions are different, so there should be a particular mathematical model for a particular region.
2. The role of cross immunity in the control of Cutaneous Leishmaniasis.
